# Giant Coronary Aneurysms

**DOI:** 10.1016/j.jaccas.2024.103222

**Published:** 2025-02-26

**Authors:** Ahmad T. Safdar, Joseph Sipko, Benjamin Yang, Tom Kai Ming Wang, Juan Pablo Umana, Michael Faulx

**Affiliations:** a Community Care Institute, Cleveland Clinic Foundation, Cleveland, Ohio, USA; b Heart, Vascular and Thoracic Institute, Cleveland Clinic Foundation, Cleveland, Ohio, USA; c Heart, Vascular and Thoracic Institute, Cleveland Clinic Weston Hospital, Weston, Florida, USA

**Keywords:** coronary angiography, coronary artery bypass, coronary circulation, left-sided catheterization

## Abstract

We report the case of a woman in her 20s presenting with 1 year of intermittent chest pain and exertional dyspnea found to have multiple giant coronary aneurysms. The largest aneurysm was found to be 7.2 × 5.2 cm in the left anterior descending artery. Because of the size and location of the aneurysms, surgical intervention was pursued and involved resection of the coronary aneurysms with reconstruction and bypass grafting.

## Graphical Abstract

**Figure:**
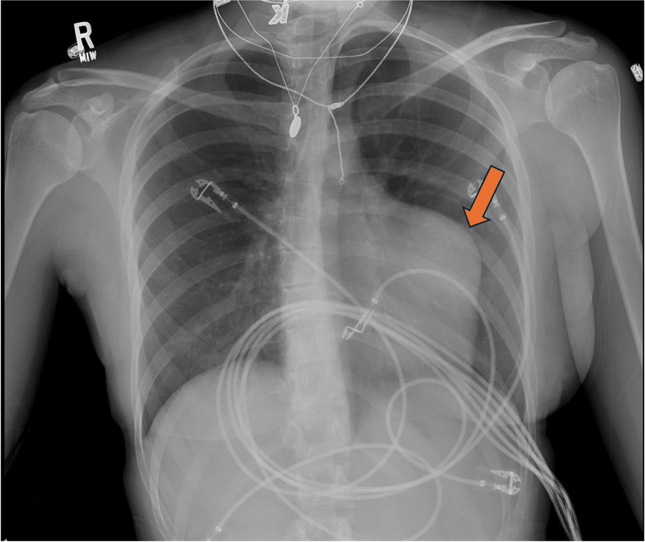

## History of Presentation

An Ecuadorian woman in her 20s with a past medical history of median arcuate ligament syndrome presented with 1 year of intermittent chest pain and progressive exertional dyspnea. She was evaluated by a cardiologist in Ecuador. Laboratory work was unremarkable aside from elevated D-dimer. She underwent a computed tomography angiography (CTA) of the chest, abdomen, and pelvis. The scan reportedly revealed several coronary aneurysms. She then presented to our hospital for further management. She denied history of recent viral illnesses, dermatologic rashes, night sweats, or headaches. She denied any recent vaccinations within the past year. She had no history of drug use. Her parents recalled an episode when she was a child of high fever lasting more than 5 days, red, swollen tongue, and peeling skin on her fingers. She recovered without intervention. She was afebrile, with a heart rate of 87 beats/min, blood pressure of 121/71 mm Hg and oxygen saturation of 100% on room air. Laboratory work including complete blood count, comprehensive metabolic panel, erythrocyte sedimentation rate, C-reactive protein, and antinuclear antibody tests were all unremarkable.

**Take-Home Messages**

• Multimodal imaging is essential for evaluating giant coronary aneurysms.
• In the absence of guidelines, multidisciplinary collaboration is key to managing coronary aneurysms.

**Figure 1:**
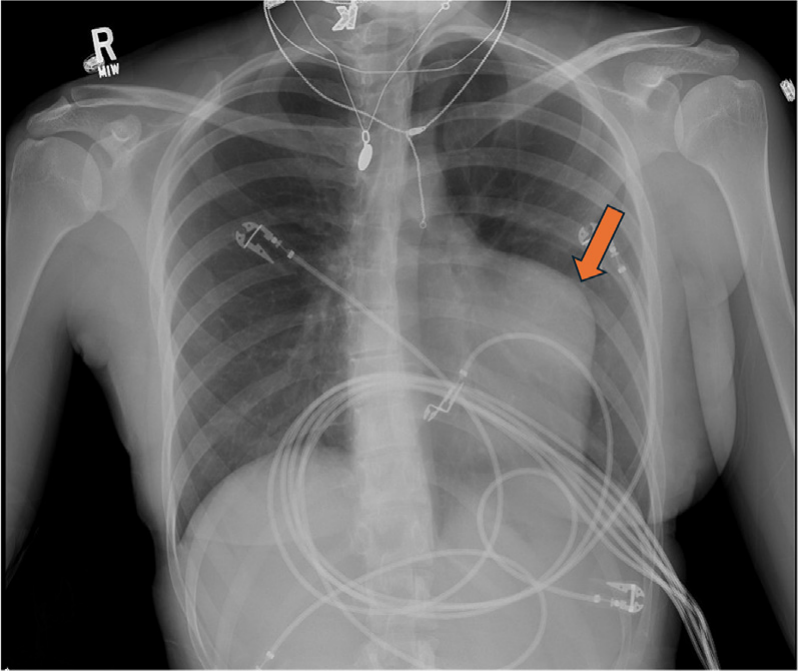
Chest X-ray Showing Abnormal Contour of Left Heart Border, Which Corresponds to Aneurysm of the Left Anterior Descending Artery Seen on Computed Tomography Angiography Chest

**Figure 2:**
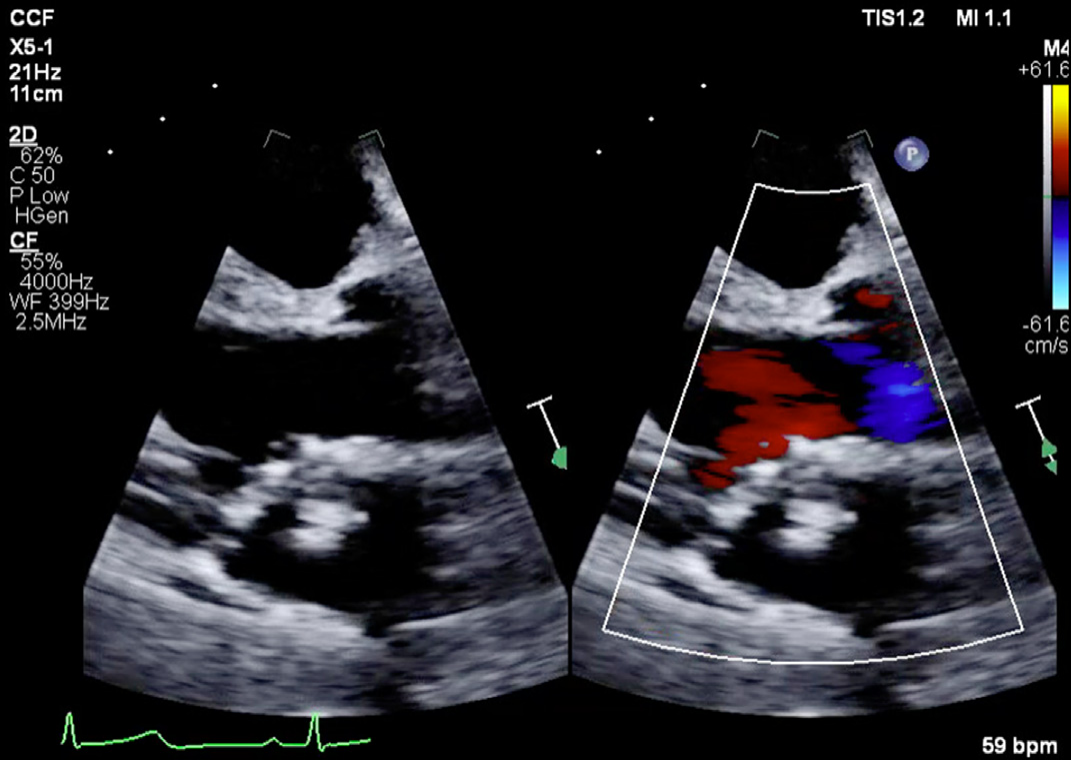
Left Atrial Echodensity (Measuring 2.3 × 2.0 cm) Was Seen and Correlated With Part of the Known Coronary Aneurysms

**Figure 3:**
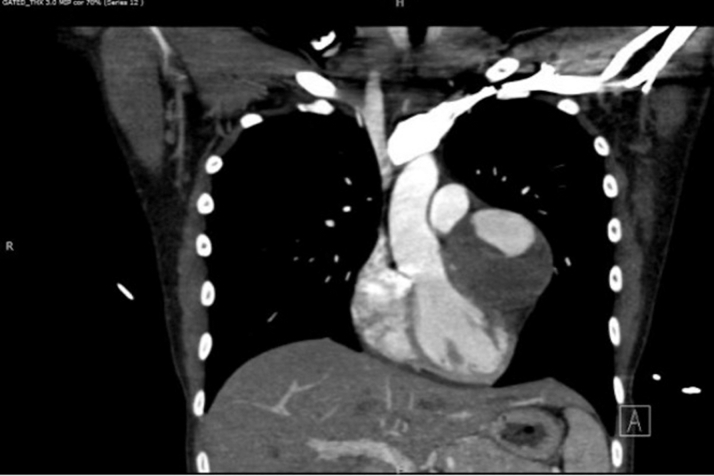
Computed Tomography Angiography Showing Proximal Left Anterior Descending Artery Aneurysm With Large Intramural Thrombus

Chest X-ray demonstrated an abnormal contour of the left heart border as seen in Figure 1. A transthoracic echocardiogram was performed that did not reveal any significant valvular disease. Left ventricular dilation was noted with an ejection fraction of 56% and otherwise normal biventricular function. A left atrial echodensity (measuring 2.3 × 2.0 cm) was seen and correlated with part of the known coronary aneurysms (Figure 2). An aorta-gated CTA of the chest, abdomen, and pelvis was repeated and revealed a 7.2 × 5.2-cm aneurysm in the proximal left anterior descending artery (LAD) with large mural thrombus (Figure 3), 3.8 × 3.1-cm aneurysm in the distal LAD with large mural thrombus and 2.7 × 1.4-cm rim calcified aneurysm in the proximal left circumflex artery (LCX) with mural thrombus. No additional aneurysms were identified. Coronary angiography was then pursued for further care planning. Findings were similar to the gated CTA. Video 1 shows 2 large coronary aneurysms involving the LAD with total occlusion (100%) and the LCX with severe obstruction (70%). The distal aneurysm of the LAD was not visible on the study because of proximal total occlusion of the LAD. There was excellent collateralization of affected vessels from the right coronary artery that supply the totally occluded LAD (Video 2).

## Management

**Figure 4:**
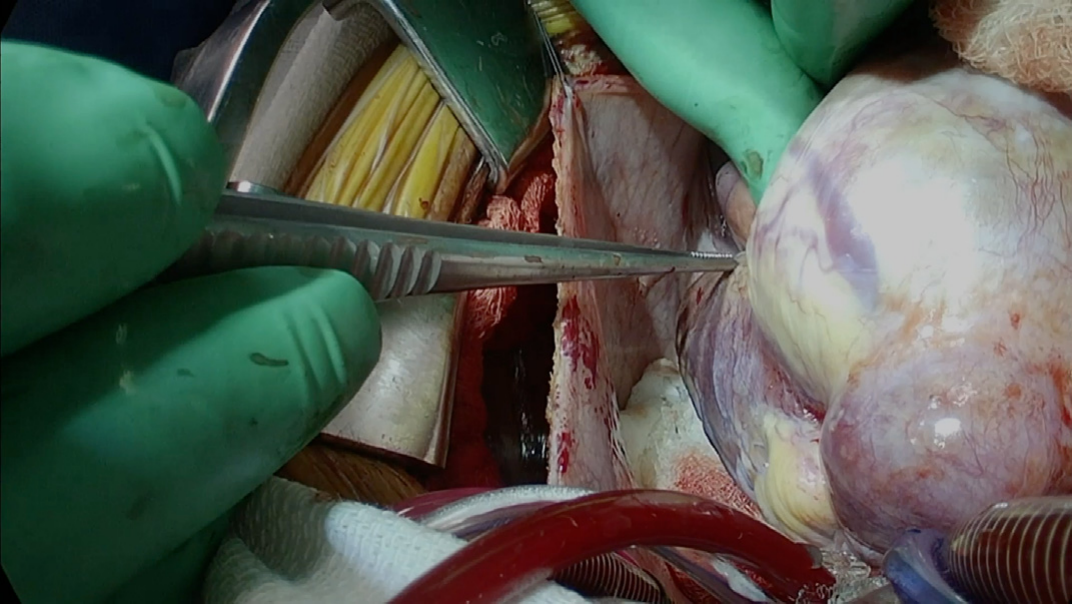
Surgical Image of Left Anterior Descending Artery Aneurysm

**Figure 5:**
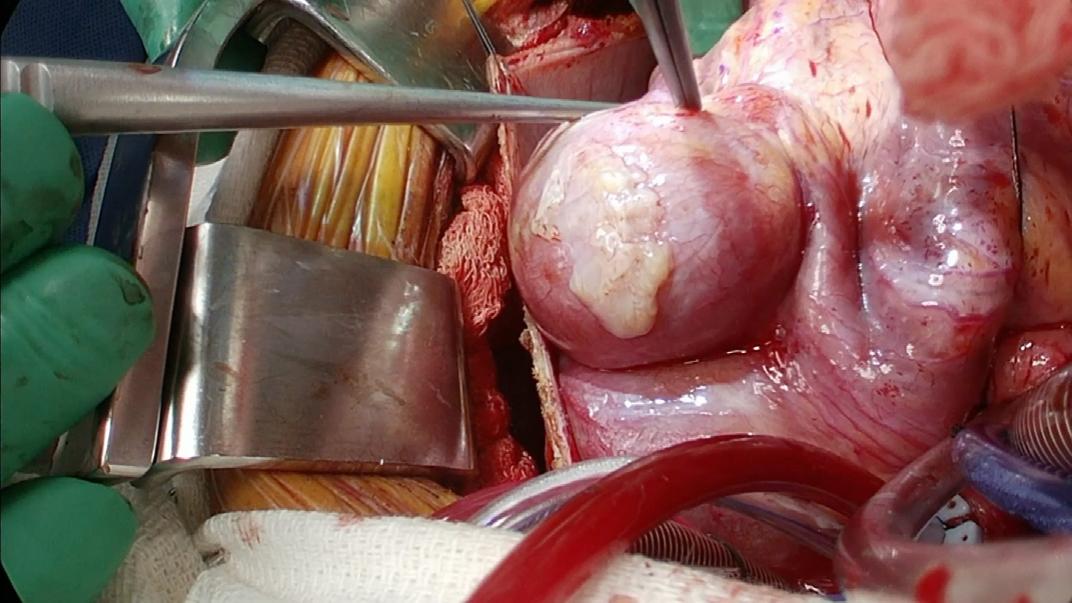
Surgical Image of Left Circumflex Artery Aneurysm

**Figure 6:**
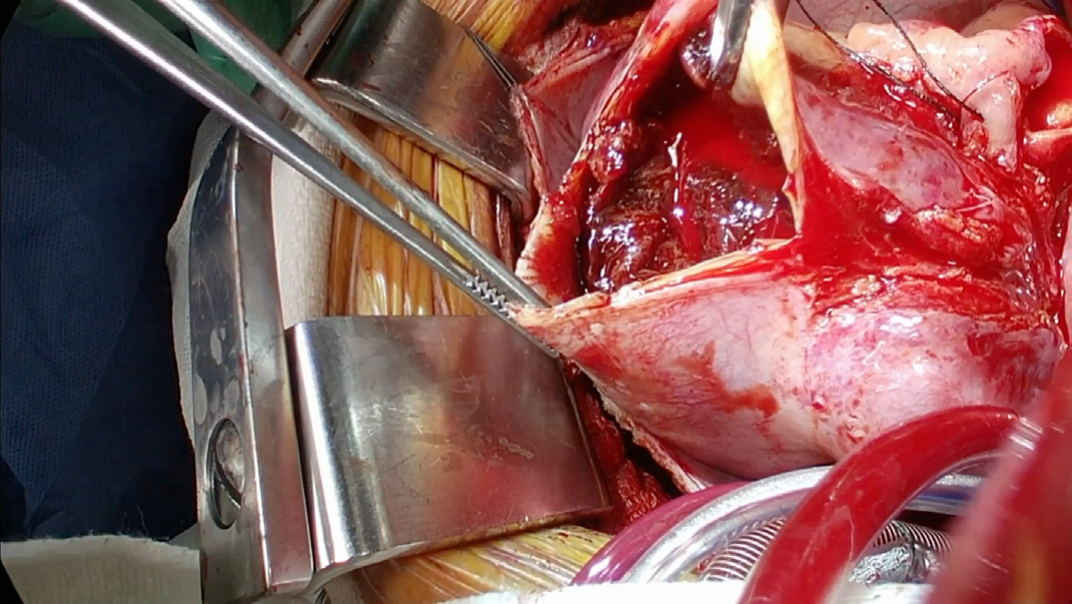
Surgical Image of Open Left Anterior Descending Artery Aneurysm With Intramural Thrombus

**Figure 7:**
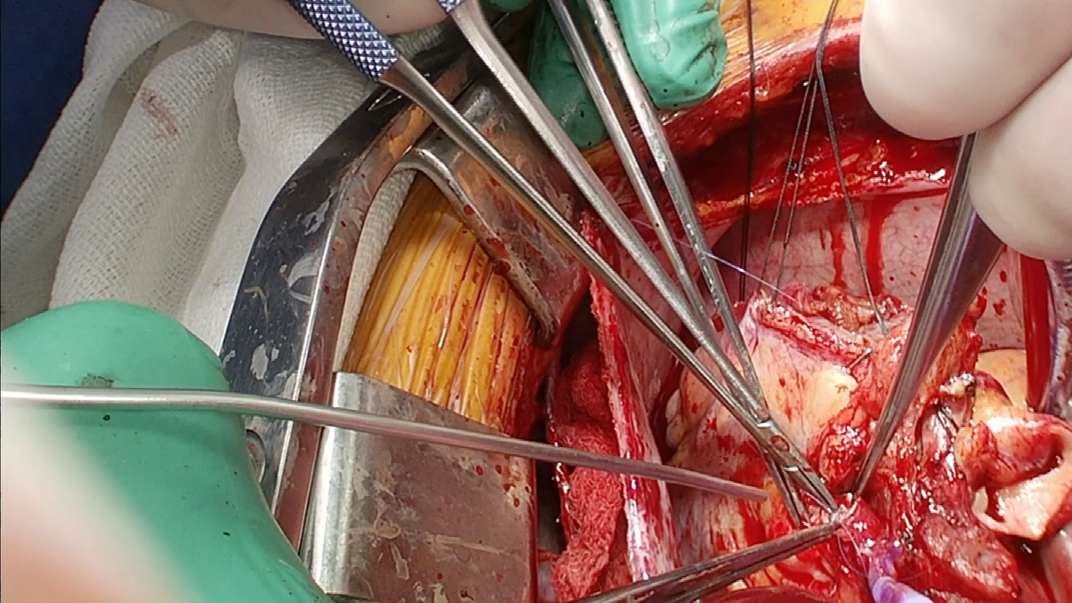
Bypass Performed to Left Circumflex Artery From the Left Main With Saphenous Venous Graft

Surgical intervention was pursued over percutaneous coronary intervention (PCI) because of the size of the aneurysms with evidence of extensive mural thrombosis. Before surgery, she was started on aspirin and therapeutic anticoagulation with heparin. The patient went to the operating room for aneurysm repair and bypass grafting. In the operating room, the large coronary aneurysms were identified (Figures 4 and 5). The coronary aneurysms were opened and extensive thrombus was evacuated, as shown in Figure 6. The aneurysm cavities were cleaned out and the distal vessels identified. Small branches were oversewn. Bypasses were performed to the LCX from the left main with saphenous venous graft as shown in Figure 7. The vein was used to reconstruct the proximal circumflex for a length of approximately 1.5 cm. Although complete arterial revascularization was considered, the diagonals were too small to graft with an arterial segment. Diagonals 1 and 2 were then bypassed in sequential fashion from the left main with the saphenous venous graft, and the distal LAD was bypassed with a pedicled left internal thoracic artery. Surgical pathology specimens were sent for the LCX and LAD clots, which revealed organized thrombus. The aneurysm wall revealed fibrous and granulation tissue.

## Outcome and Follow-Up

Postoperatively she did well and was discharged 6 days after surgery. Medications at discharge included aspirin 81 mg daily, atenolol 12.5 mg daily, Crestor 20 mg daily, and colchicine 0.6 mg for 21 days. She presented 4 months later to the emergency department with nonspecific chest pain. She underwent coronary angiography that showed patent grafts with chronically occluded proximal LAD. Her chest pain resolved during admission and she is currently doing well with no activity limitations. The plan is for her to follow up with the cardiac surgeon in 3 months with an echocardiogram.

## Discussion

Coronary artery aneurysm (CAA) describes an abnormal dilatation of a coronary artery segment, defined as 50% larger in diameter than adjacent normal segments.^1^ A universal definition of giant CAA does not exist, but diameters 20 to 50 mm have been proposed in the literature. The prevalence of giant CAA is reported to be 0.02% to 2%.^2^ Patients are usually asymptomatic with incidental discovery of the CAA. Symptoms may include angina pectoris, arrythmias, myocardial infarction, congestive heart failure, or additional complications including fistula formation, pericardial tamponade, or compression of surrounding structures. In terms of etiology, atherosclerotic disease remains the most common cause. Atherosclerosis leads to enzymatic degradation of elastic fibers of the tunica media, particularly by matrix metalloproteinases, weakening the vessel wall and predisposing to aneurysmal dilation.^3^ Kawasaki disease, vasculitis (eg, Takayasu arteritis and polyarteritis nodosa), cocaine abuse, syphilis, and trauma are less prevalent causes of CAA. Kawasaki disease causes endothelial damage from acute necrotizing arteritis, subacute/chronic vasculitis, luminal myofibroblastic proliferation, and autoimmune mechanisms such as antiendothelial cell autoantibodies.^4^ Slow flow within the aneurysm may lead to thrombus formation. The etiology of her giant CAA is unclear but is suspicious for childhood Kawasaki disease given the history provided by her parents.

Laboratory testing is used to evaluate the cause of CAA. Inflammatory markers (eg, erythrocyte sedimentation rate and C-reactive protein), antibody testing, and infectious workup are essential. Troponin testing and electrocardiogram are useful when patients present with concerns for acute coronary syndrome. Diagnosis of coronary aneurysms relies on imaging. Invasive coronary angiography provides real-time visualization of the coronary lumen and has been considered the gold standard for coronary artery imaging. High-quality imaging can be difficult during angiography due to contrast stasis in the dilated coronary segment, delayed antegrade contrast filling, and segmental back flow.^5^ Coronary computed tomography angiography allows for more accurate anatomical assessment in terms of aneurysm size and degree of thrombus than invasive angiography.^6^^,^^7^ Given her unclear disease at first, an aorta-gated CTA was ordered, instead of a coronary-gated CTA. The latter would have provided valuable aneurysm reconstructions. Nonetheless, our case demonstrates another advantage of computed tomography, as it was able to identify a distal LAD aneurysm that was missed on angiography due to proximal total occlusion of the LAD. Magnetic resonance imaging angiography is rarely used for coronary aneurysm assessment, as the spatial resolution for direct coronary artery imaging is inferior to coronary computed tomography angiography.^8^

Management of CAA remains a significant challenge, as recommendations are based on anecdotal evidence or case series. Atherosclerosis is the leading cause of CAA and aggressive risk factor modification is essential. The role of antiplatelet agents and antithrombotics is under substantial debate, particularly in patients with incidental CAA. There is a lack of high-quality studies that demonstrate any benefit or harm from antiplatelet or anticoagulant regimens.^7^ Retrospective studies suggest that angiotensin-converting enzyme agents may play a role in slowing the progression of CAA.^9^ Statins exhibit anti-inflammatory and endothelial function-improving properties, such as inhibiting the production of proinflammatory cytokines that lead to vascular damage. The American Heart Association suggests empirical treatment with statins may be considered for Kawasaki disease patients with past or current aneurysms.^10^ A short course of colchicine was prescribed to our patient to avoid post-pericardiotomy syndrome. Although colchicine’s anti-inflammatory properties in cardiovascular diseases like pericarditis have been extensively studied, its role in preventing recurrence or managing CAA is unknown.

Although there are no guidelines on PCI vs surgery, common practice for small aneurysms or high-risk surgical patients is PCI. Surgical intervention is pursued for large aneurysms and/or increased risk of rupture. There are limited data on outcomes after PCI in patients with CAA. Most of the data stems from outcomes in symptomatic patients after presenting with acute coronary syndrome. In patients with acute myocardial infarction, the critical goal is to restore flow. It is known that PCI of an aneurysmal culprit vessel in the setting of myocardial infarction is associated with low procedural success, higher incidence of no-reflow, and distal embolization, mainly due to the presence of a substantial thrombus burden.^7^ Surgical intervention for CAA may include aneurysm ligation or resection. The most common intervention is to open the CAA, remove the thrombus if present, suture the afferent and efferent vessels, and bypass grafting.

Further research is needed to understand if antiplatelet and/or anticoagulation may contribute to primary or secondary prevention of thrombotic complications. In addition, a well-developed study focused on categorizing patients in terms of symptoms, size of aneurysm, and management type (PCI vs surgery) is needed to clarify which management style should be pursued for CAAs.

## Conclusions

CAAs are often incidental findings, but rarely can manifest with exertional dyspnea or chest pain. Complications of CAA can lead to sudden death from myocardial infarction or rupture of the affected artery. Current management is based on anecdotal evidence or case series and future comparative trials are essential to define the appropriate management strategy in patients with CAA.

## Funding Support and Author Disclosures

The authors have reported that they have no relationships relevant to the contents of this paper to disclose.

## Appendix

Video 1 Coronary Angiography Showing LCX Aneurysm, LAD Aneurysm, and Distal Chronic Total Occlusion of LAD

Video 2 Coronary Angiography Showing Collaterals From the Right Acute Marginal Branch That Supplies the Distal LAD Territory A small aneurysm of the distal collateral is seen.
